# Dr. Bone on Yellow Fever, &c.

**Published:** 1847-04

**Authors:** 


### Art. VII.

*Inaugural Dissertation on Yellow Fever, and on the Treatment of that Disease by Saline Medicines.* By G. F. Bone, m.d., Assistant Surgeon to the Forces. With an Appendix, *On the Principles to be observed in providing Barracks and Hospitals for Troops in the West Indies.* By Hugh Bone, m.d., Inspector-General of Army Hospitals.—*Edinburgh,* 1846. 8vo, pp. 85.

Inaugural dissertations, generally speaking, have a very characteristic amount of schoolboy orthodoxy about them. It cannot be expected of them to contain much practical matter gleaned from experience; and it is only rarely that the student adventures any original investigations that are other than crude, or superfluous, or commits himself to research deeper than that of culling facts and phrases from the leading text-books of the day. We should, from internal evidence, have inferred that the pamphlet before us had a collegiate origin: it is so redolent, in places, of Hippocrates, Celsus, Gregory, and practice of physic-lectures. But, beyond these graduation merits, it possesses others of a different stamp, for which the author is indebted to the practical experience and judgment of his father. We cannot help thinking, however, that Dr. Bone, sen. would have done himself more justice, and medical science more service, had he presented a monograph on yellow fever to the profession, altogether his own production, and embodying the results of his own practice, instead of confiding the task to one who is manifestly a very inexperienced writer. Though there are no less than twenty "*corrigenda*" in this *brochure* of eighty-five pages, there are many sins against literary taste and skill that still remain uncancelled; and many of these faults not easily to be overlooked in the present age of literary and technical precision.

Dr. Bone's definition of yellow fever occupies eleven lines and a half. He says, "it is not brief, and is perhaps redundant, but it is reciprocal (?) with the disease defined, for whoever has these symptoms has yellow fever, and if I have failed in making this definition, I have failed in a bold attempt, for neither Dr. Good, nor Dr. Jackson attempted to define yellow fever." We are afraid Dr. Bone has not read these authors with the care that is due to them. He gives Dr. Good the precedence, though Jackson's work on fever appeared some twenty-four years before the first edition of Good's 'Study of Medicine'. Now, if Dr. Bone will take the trouble of turning to page 179 of Jackson on Fever, edit. 1798, and over the rest of the pages intervening between this and page 205, he will find that they are all occupied with definitions of the disease. Twenty-six pages of definition, and not moderately minute, Dr. Bone considers nothing, whilst eleven lines and a half of his own he esteems redundant! As regards Good's account of the symptoms of yellow fever, if Dr. Bone will consult pages 635, 641 et seq. of the first volume of the last edition of the 'Study of Medicine,' he will find definition enough, at least in comparison with his own.

Dr. Bone believes that yellow fever is, under all circumstances, non-contagious; and he gives some striking illustrations of the fact:

"It is not the diluting breeze that stops the spread of yellow fever, but the removal from foul air, whether stagnant or rushing with great force. That this plan is successful, is proved by the following examples among many that might be quoted. The white troops in St. Kitt's were removed from Brimstone Hill and encamped on healthy ground in August 1840, and in February 1843, and in July 1844, and uniformly the yellow fever stopped making progress among them. The white troops, except 13 men of the Royal Artillery, were brought from St. Kitt's to Barbados in August 1844, and did not import the disease into Barbados. The 33d regiment were moved from the stone-barrack, Barbados, to Gun-hill encampment ground, on the 3d December, 1841, and immediately became healthy." (p. 18.)

"Fifty of the patients were yellow, and eleven of them died, and were all carefully dissected by him (Dr. Bone, sen.) or by his assistants, yet none of them caught yellow fever, nor any of the other patients in the quarantine hospital, nor any of the hospital servants or washerwomen, nor any of the patients or servants in the hospital from which the yellow patients were taken. He calculated that 700 persons had been exposed to the influence of the disease, yet none caught yellow fever. The cordon of troops did their duty, the British strictly, the Spanish with ferocity, but could not prevent all intercourse with the quarantine hospital. One evening, when one of his assistants, Mr. Williams, assistant surgeon, 23d regiment, made his visit to the female ward, he discovered that one of the female patients had her sweetheart, a sergeant from the depôt, in bed with her. She was then yellow as an orange, and the disease was running its course, but the sergeant did not catch yellow fever. There was in the same ward another female who contrived to see her sweetheart while she was affected with the disease, and he did not catch yellow fever." (pp. 19, 20.)

A still more striking case illustrative of the incommunicableness of yellow fever, than any Dr. Bone has given, is recorded by Moreau de Jonnès. A soldier named Guyon, of Fort Royal, Martinique, had the filthy hardihood to wear for twenty-four hours, a shirt completely saturated with the sweat of a yellow fever patient suffering from the worst form of the disease; he was then inoculated in each arm with the matter discharged from suppurating sores; he slept for six hours and a half in a bed soiled by the excrement, and stained by the sweat of a patient who had just died in it; and consummated a variety of other similar brutalities, by drinking a couple of ounces of the black fluid taken from a dead man's stomach. This man or beast, however, did not take the fever.

All this may be true, and no doubt is true of the ordinary yellow fever of the West Indies and coast of Africa; and yet yellow fever may be contagious for all that—at certain times and in certain modified forms. There can scarcely be a doubt that in all the ordinary localities of the non-contagious yellow fever, a contagious epidemic yellow fever *does* sometimes show itself, presenting, for the most part, the ordinary phenomena—only in an aggravated degree, and springing, in the first instance, apparently, from the ordinary sources. Whether, in such a case, we have to deal with an entirely different disease—*a nova pestis,* or merely with a modification or *degeneration* of the old,—is a question yet unsolved. We greatly desiderate a true discriminating history of *all* the maladies—endemic and epidemic—that have ravaged our colonies, and our fleets and armies, under the one name of yellow fever.

In the *treatment* of yellow fever, Dr. Bone is strongly averse to the practice of many of his predecessors. Dr. Rush, regarding all remittent fevers within the tropics as dependent on local inflammation or congestion, used the lancet unsparingly, and (as he thought) with the best effects. Jackson followed his plan, and with like success. Dr. Bone says, "my experience during many years does not confirm this notion. In 1820, in Tobago, I employed the bleeding practice: it failed totally, and since then I discourage the abstraction of blood." (p. 46.)

Even local depletion he disapproves of.

> "Leeching the temples, cupping the nape of the neck, &c., are not necessary measures, as may be understood from the observations already made. The cause of the headache is in the abdomen, and only secondarily or by sympathy, in the brain; it is therefore to be cured by acting principally on the bowels by purgatives, and on the skin by diaphoretic medicines; but leaves applied to the brow to excite perspiration are useful, and the hands and feet may be placed in hot water." (p. 47.)

Mercury was once so favorite a remedy in yellow fever, that Chisholm said of it—"Let it never be forgotten, that, at whatever period of the disease salivation is excited, whether the supposed signs of putrefaction have appeared or not, the accession of it is the certain signal of cessation of disease, and of returning health." This opinion has had many able supporters, even up to the present day; but Dr. Bone entirely disapproves of it. "Salivation," he says, "neither cures nor prevents yellow fever, but, on the contrary, by rendering the body more sensible to impressions from currents of cold air, *predisposes* to yellow fever." (p. 48.)

Dr. Bone lays great stress upon the use of salines, and saline aperients in this disease. Seidlitz powders, Rochelle, Cheltenham, and Epsom salts, cream of tartar, and such like, he chiefly recommends.

> "During the first twenty-four hours, the aperients and diluents are to be assiduously and skilfully urged until twelve or twenty stools are procured, or until *the stools become nearly the fluids drunk*; and usually from two to four ounces of the neutral aperient salts, and from one to two gallons of diluent liquors, are about the average quantities necessary to produce a solution of the disease." (p. 39.)

This is diluent, deobstruent, and solvent practice with a vengeance. It may possibly be not so injurious as the bloodletting and mercurial heroisms; but we should ourselves have greatly preferred the "one to two gallons of diluent liquors" *minus* the hydragogue accompaniments.

The Appendix contains some very judicious, and seemingly practicable suggestions, for "providing hospitals and barracks for troops in the West Indies."

